# Efficient incremental training using a novel NMT-SMT hybrid framework for translation of low-resource languages

**DOI:** 10.3389/frai.2024.1381290

**Published:** 2024-09-25

**Authors:** Kumar Bhuvaneswari, Murugesan Varalakshmi

**Affiliations:** ^1^School of Computer Science Engineering and Information Systems, Vellore Institute of Technology, Vellore, Tamil Nadu, India; ^2^School of Computer Science and Engineering, Vellore Institute of Technology, Vellore, Tamil Nadu, India

**Keywords:** hybrid NMT-SMT, incremental training, beam search, SMT phrase table, low-resource languages

## Abstract

The data-hungry statistical machine translation (SMT) and neural machine translation (NMT) models offer state-of-the-art results for languages with abundant data resources. However, extensive research is imperative to make these models perform equally well for low-resource languages. This paper proposes a novel approach to integrate the best features of the NMT and SMT systems for improved translation performance of low-resource English–Tamil language pair. The suboptimal NMT model trained with the small parallel corpus translates the monolingual corpus and selects only the best translations, to retrain itself in the next iteration. The proposed method employs the SMT phrase-pair table to determine the best translations, based on the maximum match between the words of the phrase-pair dictionary and each of the individual translations. This repeating cycle of translation and retraining generates a large quasi-parallel corpus, thus making the NMT model more powerful. SMT-integrated incremental training demonstrates a substantial difference in translation performance as compared to the existing approaches for incremental training. The model is strengthened further by adopting a beam search decoding strategy to produce *k* best possible translations for each input sentence. Empirical findings prove that the proposed model with BLEU scores of 19.56 and 23.49 outperforms the baseline NMT with scores 11.06 and 17.06 for Eng-to-Tam and Tam-to-Eng translations, respectively. METEOR score evaluation further corroborates these results, proving the supremacy of the proposed model.

## 1 Introduction

In recent years, NLP has gained popularity due to the growing quantity of text and speech data. A few of the common NLP tasks are machine translation, text classification, text extraction, and natural language generation. In today’s globally interconnected world, machine translation is indispensable for producing automatic, fast, and error-free translations. Contemporary machine translation systems such as statistical machine translation (SMT) and neural machine translation (NMT) are heavily reliant on the amount and quality of training data. Deep learning has revolutionized NLP by enabling the data-driven neural networks to harness the exponentially growing data and achieve cutting-edge performance for a variety of NLP tasks.

Availability of large parallel corpus is the significant driving force for the emergence of neural machine translation (NMT) (Koehn et al., 2003). NMT achieves very promising results on a variety of language pairs (Bahdanau et al., 2014), with sequence to sequence (seq2seq) being the most widely used NMT model (Sutskever et al., 2014). The encoder and decoder are the two main components of the seq2seq model, where the encoder learns to transform variable-length sentences of the source language, while the decoder learns the target sentences of the destination language as output (Lamb and Xie, 2016). The memory consumption of NMT model is significantly less and works faster than that of conventional SMT models and yields promising results for high-resource language pairs (Cho et al., 2014).

For some languages like Indic languages, there are not enough resources to train a robust NMT system. Tamil is one of the longest-surviving classical languages of India and has the oldest extant literature among Dravidian languages from around the 3rd century BC (Tamil Language, 2023). A total of 88.6 million people speak Tamil worldwide. Building an NMT for a non-English language like Tamil requires an enormous amount of data to train and claim good results. However, the publicly available data for English–Tamil parallel corpus are very much limited.

In the literature, monolingual data have proved to increase the corpora size of the low-resource languages, thereby enhancing the quality of machine translation models for those languages (Gulcehre et al., 2015). Monolingual data are usually much easier to obtain, more diversified, and have been attractive resources. Back-translation is an intriguing technique for utilizing monolingual data in NMT. Using the original NMT model to translate monolingual sentences from source language to target language (He et al., 2019; Jiao et al., 2021) and a reverse translation model to translate monolingual sequences from target-to-source language (Edunov et al., 2018; Fadaee et al., 2017) results in producing synthetic parallel sentences. However, the low quality of the synthetic parallel data generated from monolingual sentences is a serious impediment to further advancement of back-translation, despite the fact that it has been shown to be reliable and efficient.

Based on the gaps identified in the literature, this research formulates two major objectives—(1) explore better and efficient ways to utilize the monolingual corpus; and (2) build a more powerful translation model to work in low-resource settings.

The first objective can be achieved with incremental training, which has drawn the attention of the research community as an alternative way to make the best use of monolingual data (Singh et al., 2016). The model trained with only the best translations of the monolingual corpus in each iteration will improve the translation quality in successive iterations. Incremental training generates high-quality quasi-parallel data.

Towards attaining the second objective, a comparative analysis of NMT and SMT models is performed. It reveals that NMT unarguably produces fast and more fluent translations and works well for languages with different word order, but if the parallel corpus volume is less and the data quality is poor, SMT is still a more viable option.

Existing literature provides evidence to show that phrase-based statistical MT (PB-SMT) has been the crux of machine translation for more than two decades. SMT ensures that every word in the source phrase is translated to a semantically related target (Wang et al., 2018). Comparative studies between NMT and phrase-based statistical translation model reveal that NMT cannot guarantee that all source words are properly translated, and all the words in the source sentence are aligned to words in the target sentence; but statistical machine translation translates every source word, treats words as discrete symbols, and explicitly memorizes all the translations including rare words (Almahairi et al., 2016; Wang et al., 2017). However, statistical machine translation does not work well between English and Dravidian languages like Tamil because of its rich morphological nature that has a significantly different word order and unknown words (outside of vocabulary).

As both NMT and SMT have their pros and cons, a hybrid model combining the best features of SMT and NMT can evolve as a more powerful model, thus addressing the second challenge. The model can be strengthened further by implementing a beam search decoding technique. Several studies substantiate the effect of beam search decoding in improving the translation performance of sequence-to-sequence models (Freitag and Al-Onaizan, 2017; Park et al., 2020; Yang Y. et al., 2018; Jahier Pagliari et al., 2020; Chen et al., 2019; Banik et al., 2018).

Thus, this research proposes a novel NMT-SMT hybrid framework that optimizes the baseline NMT comprising encoder–decoder with attention, by incrementally training it with the best translations of the monolingual data from both the source and target languages. An SMT phrase pair table is employed to determine the best translations among the NMT outputs, based on the maximum match between the words of the phrase-pair dictionary and each of the individual translations. Output of this optimized NMT model is decoded using a beam search technique to obtain the best possible translations for each sentence. This improves the translation performance for low-resource languages.

The rest of the paper is organized as follows. Section 2 reviews some of the relevant background literature on monolingual corpus and resource-constrained parallel languages. Section 3 elaborates on the proposed model that implements incremental training, SMT phrase-table integration, and beam search decoding. Section 4 specifies the experimental set-up and the corpora details used for the research. In detail, Section 5 discusses the results of the different model variants obtained for various categories and sizes of parallel and monolingual corpora. Section 6 draws inferences from our study and provides guidelines for future extensions of the study.

## 2 Related studies

Imamura et al. (2018) and Jiao et al. (2021) showed that synthetic parallel data generated via sampling improves the translation accuracy. Yang Z. et al. (2018) showed an improved translation performance by using two encoders instead of a single one, to map the sentences of the language pairs to a shared latent space. Sharing of weights between the encoders increases the efficiency of the model, as well. To make the model more generalized and suitable for low-resource languages, Hong-Viet et al. (2021) added noise to the output of the model trained with monolingual data of the target language. A denoising autoencoder and pre-ordering are used for this purpose. In another study related to the efficient translation of low-resource languages, Qi et al. (2018) used pre-trained embeddings. These embeddings demonstrate significant progress in multilingual training scenarios. Back-translation and integration of NMT and SMT are also explored in the literature, for better translation quality, as listed in Table 1. The proposed study aimed to address the gaps identified in the literature.

**Table 1:** Literature on back-translation and NMT-SMT integration.

Papers	Method	Gaps identified	Datasets used
Lample et al. (2018)	Back-translation of monolingual data	Experimented only in an unsupervised setting but not in a semi-supervised setting where limited parallel corpus can also be used for the initial training	WMT monolingual News Crawl datasets, WMT’16, LDC2010T21, and LDC2010T23 corpora
Sennrich et al. (2015)	Back-translation of monolingual data	Does not achieve maximum gain because of employing smaller monolingual datasets for back-translation.	WMT 15 task, IWSLT 14 task, IWSLT 15 task
Vyawahare et al. (2022), Artetxe et al. (2017), Singh and Singh (2022)	Back-translation applied in semi-supervised setting	Back-translation does not generate a high-quality parallel corpus	IndicCorp, Samanantar dataset, bilingual dataset (Madasamy et al., 2022)
Zhang et al. (2017)	Integrates NMT and SMT in which the SMT phrase-based decoding cost is used to rerank the n-best outputs of NMT model	Does not produce better results than the baseline model for a few language pairs	NTCIR-9, Europarl corpus, WMT 2014, and WMT 2015 were used for four different language pairs.
He et al. (2016)	Log-linear framework is used to integrate the translation model and an n-gram language model of the SMT with the NMT that better handles out-of-vocabulary words and significantly improves the translation quality.	Not yet explored the integration of phrase pairs with NMT which can help in idiom translation	NIST MT06 and NIST MT08
Zhang et al. (2021)	Builds a search space with readily available phrase alignment, similar to PB-SMT. This helps to explicitly introduce phrase alignment into the translation process of the NMT, so as to make the translation interpretable	Focuses on input and output alignment for high-resource languages and yet to be explored for low-resource languages	LDC corpora, NIST MT06, and NIST MT08
Zhang et al. (2018)	Both source-to-target and target-to-source NMT models are updated through several iterations concurrently (incremental training) using a joint expectation–maximization (EM) optimization technique	Translation probability of the NMT itself is used for selecting the best translations. A different and better assessment strategy should be used, to produce optimal results	LDC corpora, Gigaword corpus, NIST OpenMT 2006, NIST 2003, NIST 2005, NIST 2008, and NIST2012 news-test 2012, news-test 2013 and news-test 2014

## 3 Methodology

**Figure 1:**
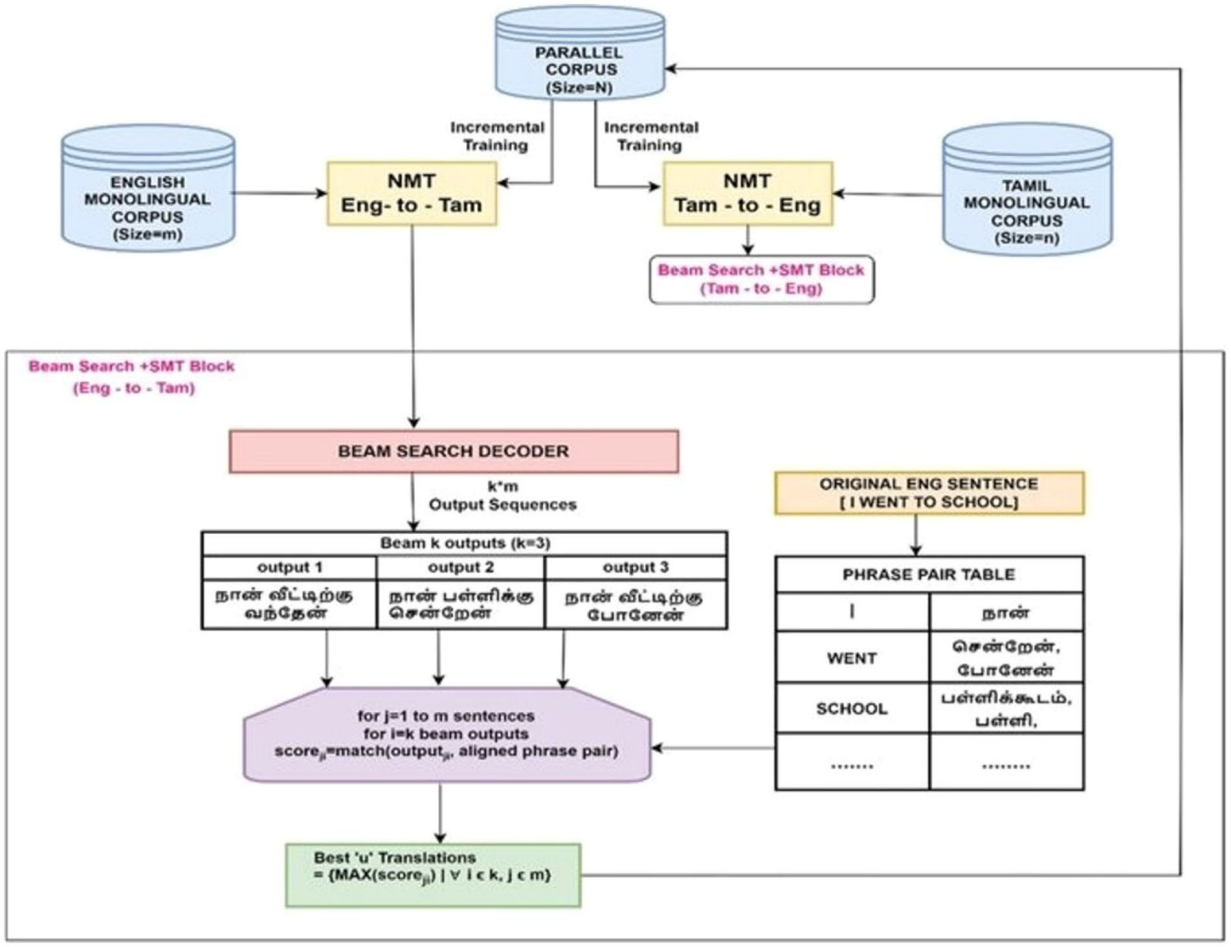
Proposed hybrid framework that incrementally trains the NMT with the best translations of the monolingual corpus as evaluated by the SMT framework and includes beam search decoding to optimize the translation performance for low-resource languages.

Incremental training is a promising technique for enhancing NMT model performance resulting in better translation quality while also saving time and resources. The proposed method further attempts to augment the benefits of incremental training using a hybrid NMT-SMT framework and beam search decoding technique. Figure 1 depicts the model architecture comprising four phases.

These four phases are designated as four different model variants.

- Model Variant I—baseline NMT
- Model Variant II—baseline NMT + random sampling-based incremental raining with monolingual corpus
- Model Variant III—baseline NMT + SMT-integrated incremental training
- Model Variant IV—baseline NMT + SMT-integrated incremental training + beam search

### 3.1 Model variant I—baseline NMT

Two NMT systems, namely Eng-to-Tam and Tam-to-Eng, are built using the encoder–decoder model with an attention mechanism, for source-to-target translation and vice versa. The small parallel corpus available for English–Tamil is pre-processed by splitting the sentences into words, removing punctuation characters, changing into lowercase, tokenizing, and then encoding them. Initially, the two NMT models are trained with the parallel corpus.

#### 3.1.1 Encoder–decoder model with attention

Seq2Seq model with an attention mechanism consists of an encoder, decoder, and attention layer. The drawback of the vanilla version of encoder–decoder architecture without attention is that the encoder must remember everything after reading the complete sentence just once before turning it into an encoded vector. Longer sentences will result in information loss because the encoder will not be able to recall the beginnings of the sequence. Instead of giving equal weightage for each word, specific words need to be emphasized more than the others. The attention mechanism is introduced to deal with these issues.

The encoder creates a sequence of hidden states from a sequence of input tokens. Following the hidden states, the decoder generates a series of output tokens. When creating each output token, the attention mechanism enables the decoder to pay attention to various elements of the input sequence. A stack of LSTM layers makes up the encoder. Each LSTM layer produces a new hidden state by using the most recent input token and the output of the previous hidden state. Another stack of LSTM layers constitutes the decoder. However, the decoder also incorporates another layer for attention. The attention layer creates a weight vector using the encoder’s hidden states and the decoder’s current hidden state. This vector is used to find the weighted sum of the encoder’s hidden states. A context vector is the outcome, and the decoder uses it to produce the subsequent output token. When creating each output token, the attention mechanism enables the decoder to pay attention to various elements of the input sequence. This is crucial as it enables the decoder to generate the subsequent output token while taking into account the context of the previous output tokens. The decoder can produce more accurate and fluent translations as a result.

The basic principle of the attention mechanism is to pay attention to the different input vectors of the input sequence depending on the attention weights, rather than attempting to learn a single-vector representation for each sentence. Using a set of attention weights, the decoder will be informed at each stage of decoding about how much “attention” must be given to each input word. The decoder for translation receives contextual information from these attention weights. Based on the context vectors linked to the source position and the previously generated target words, the model predicts the current target word.

Attention layer consists of

- Alignment score layer
- Attention weights
- Context vector

##### 3.1.1.1 Alignment score layer

The alignment score denotes how well the inputs around position “j” and the output at position “i” match. The score is based on the decoder’s previous hidden state, S_i-1_ just before predicting the target word and the hidden state, and h_j_ of the input sentence (Equation 1).


(1) $$e_{ij}=a\;\left(s_{i-1},h_{j}\right)$$

e_ij_ is the output score of a feedforward neural network described by the function ‘a’ that attempts to capture the alignment between input at *j* and output at *i*. Using e_ij_, the attention weights are determined. The e_ij_ weights are then normalized using the Softmax function to yield α_ij_. Instead of encoding the entire information of the source sentence into a fixed-length vector, the decoder chooses that portion of the source sentence, it should concentrate on. The alignment vector has the same length as the source sequence and is estimated at each decoder time step.

##### 3.1.1.2 Attention weights

The attention weights α_ij_ are computed by applying Softmax activation function to the alignment scores given by Equation 2.


(2) $$\alpha_{ij}=\exp\;\left(e_{ij}\right)/\sum_{k=1}^{x}\exp\left(e_{ik}\right)$$

Softmax activation function returns probabilities whose sum equals 1. This helps to illustrate how it influences weight for each input sequence. The greater the attention weight of the input sequence, the greater is its impact on predicting the target word.

##### 3.1.1.3 Context vector

Once all the inputs and associated weights for the decoder are available, the decoder can be constructed. The context vector is used to estimate the decoder’s final output. Equation 3 represents the mapping of the input sentence to the context vector C_i_, which is the weighted sum of attention weights and encoder hidden states (h_1_, h_2_, …, h_x_).


(3) $$C_{i}=\sum_{j=1}^{x}\alpha_{ij}\;h_{j}$$

To predict the target word, the decoder uses context vector (Cᵢ), decoder’s output from the previous time step (Yᵢ−₁), and decoder’s previous hidden state (Sᵢ−₁). The context vector C_i_ is fed to the decoder LSTM, which decodes the next possible word’s probability distribution. This decoding operation applies to all time steps present in the input, as given in Equation 4.


(4) $$S_{i}=f\;\left(S_{i-1},C_{i},Y_{i-1}\right)$$

### 3.2 Model variant II—baseline NMT + random sampling-based incremental training with monolingual corpus

The suboptimal NMTs (model variant I) trained with the small parallel corpus of size *N*, in the previous phase, are improved for better translation performance using incremental training. It starts with pre-processing the two monolingual corpora. As explained in Algorithm 1, the pre-processed input sequences in the English monolingual corpus are grouped into ‘a’ individual batches with each batch containing an optimum number of randomly sampled input sequences, *p*. Similarly, the sentences in Tamil monolingual corpus are grouped into ‘b’ individual batches with each batch containing an optimum number of sentences, *q.* ${\left\{{\left\{e^{ij}\right\}}_{j=1}^{p}\right\}}_{i=1}^{a}$ and ${\left\{{\left\{t^{ij}\right\}}_{j=1}^{q}\right\}}_{i=1}^{b}$ represent the two monolingual corpora. In the first iteration, the suboptimal Eng-to-Tam NMT model predicts for the first batch of English monolingual sentences, ${\left\{e^{j}\right\}}_{j=1}^{p},$ and simultaneously, the Tam-to-Eng NMT model predicts for the first batch of Tamil monolingual sentences, ${\left\{t\right\}}_{j=1}^{q}$. The source sentences and the predicted sentences of both the models contribute to a total of *p + q* quasi-parallel sentences, ${\left\{e^{j},{\hat{t}}^{j}\right\}}_{j=1}^{p}+{\left\{t^{j},{\hat{e}}^{j}\right\}}_{j=1}^{q}$, which are added to the original bilingual corpus. The two models are retrained with the increased parallel corpus, and the second iteration is performed with the second batch of monolingual sentences. The generated quasi-parallel sentences are appended to the parallel corpus, and this process repeats iteratively until all the batches in the monolingual corpora are used up to build a large quasi-parallel corpus. The translation performance of the two NMT models increases incrementally, as the models are retrained with the quasi-parallel sentences generated in each iteration.

#### ALGORITHM 1 Incremental training.


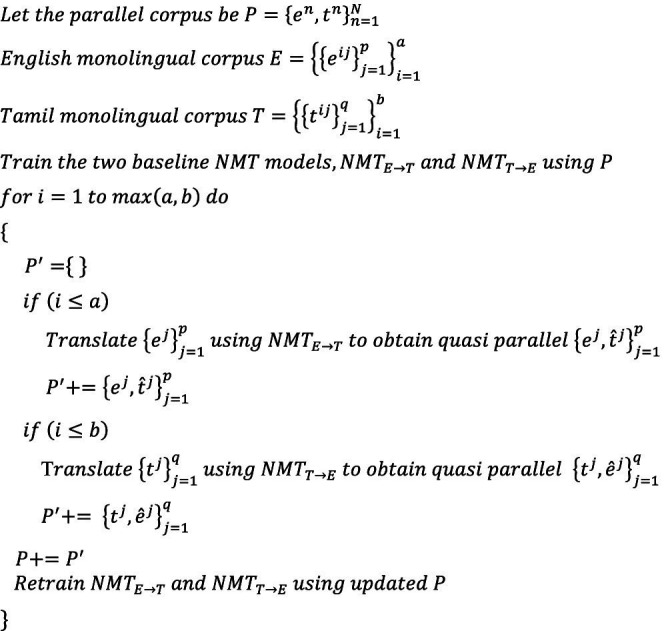


### 3.3 Model variant III—baseline NMT + SMT-integrated incremental training

Existing studies on incremental training either use batches of randomly sampled monolingual data or use the best translations identified using the translation probability of NMT, to retrain the NMT. It is less likely that the probability estimates of a suboptimal NMT identifies the “true” best translations. Undoubtedly, using a better assessment strategy that can identify the “true” best translations will amplify the power of incremental training manifold. From the literature, it is evident that SMT unlike NMT does not expect a large amount of higher quality, in-domain training data for high translation performance. Hence, the phrase table generated as a part of SMT can be reliably integrated with incremental training to determine the best translations.

GIZA++, an open-source and easy-to-use tool is used to construct the phrase table from the parallel corpora. It generates high-quality word alignments. Majority of modern SMT systems employ GIZA++, a generative model, to automatically align words from the sentence-aligned parallel corpora. Let E be a source-language sentence with tokens (e_1_, e_2_, …, e_m_) and T be a target-language sentence with tokens (t_1_, t_2_, …, t_n_). Alignments are defined as


(5) $$A\subseteq \left\{\left(e,t\right)\in E\times T\right\}$$

where each *e*, and *t* are translations of one another (Equation 5). Performance is typically measured with alignment error rate (Och and Ney, 2000). GIZA++ works well when large sentence-aligned corpora are used. Table 2 lists a few sample phrase pairs generated along with the alignment score using GIZA++.

**Table 2:** Sample phrase pairs generated with their equivalent alignment scores using GIZA++.

# Sentence pair (1) source length 3 target length 3 alignment score: 0.0024918 Are you happy? 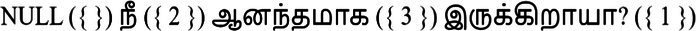
Sentence pair (2) source length 2 target length 3 alignment score: 0.0044181 Are you Ravi? 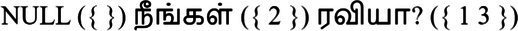
Sentence pair (3) source length 4 target length 5 alignment score: 8.5551e-06 Was the coffee very hot? 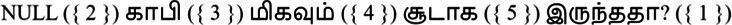
Sentence pair (4) source length 3 target length 4 alignment score: 2.21761e-05 Do you speak Hindi? 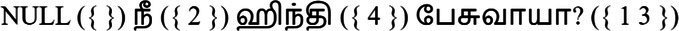
Sentence pair (5) source length 4 target length 7 alignment score: 3.20197e-08 Should I come to the temple 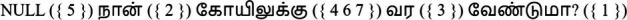

Using the phrase pairs generated using GIZA++, a dictionary, *eng_tam_dict* as shown in Table 3 is built with English word as the key and the list of Tamil words with equivalent meaning, as the value. Algorithm 2 explains how the phrase table is employed to evaluate the scores of the translated sentences. The *eng_words* list contains the words of the original English sentence to be translated. For each of these English words, the phrase-pair dictionary is looked up to find the list of equivalent Tamil words. The individual Tamil words in this list are compared with the words of the output sentences. For each Tamil word in the phrase pair that matches the words of the output sentence, the score of an output sentence is incremented by 1. As stated in Algorithm 3, the output sentences are sorted in the decreasing order of their scores and the first *u* translations are appended to the parallel corpus. Table 4 lists three sample English sentences and illustrates the score evaluation for the corresponding model outputs, based on the mappings of the *eng_tam_dict* dictionary, and highlights the two best translations out of three.

**Table 3:** *eng_tam_dict* dictionary constructed from phrase pairs of SMT.

‘I’	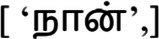
‘went’	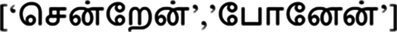
‘school’	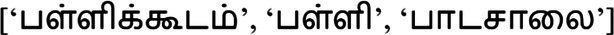
‘happy”	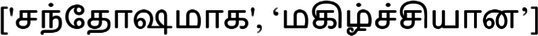
‘classroom’	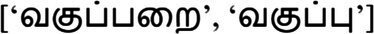
‘came’	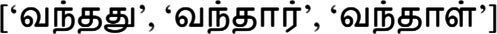
‘teacher’	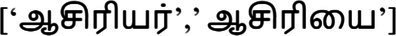
‘wrote’	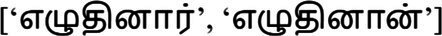
‘ram’	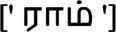
‘a’	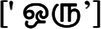
‘he’	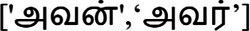
‘story’	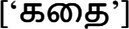
‘very’	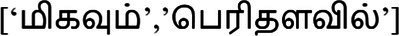
‘told’	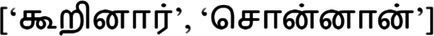
……………	……………………………

**Table 4:** Score calculation for three sample sentences to select the best two translations.

English sentence	Tamil translated sentences	Score	Best two translations
1. I went to school	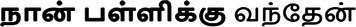	2/3 = 0.66	Sentences 1 and 2
2. He told a story	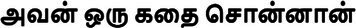	4/4 = 1	
3. Teacher came to the classroom	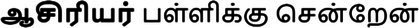	1/3 = 0.33	

#### ALGORITHM 2 Score calculation for output sentences by integrating SMT results.


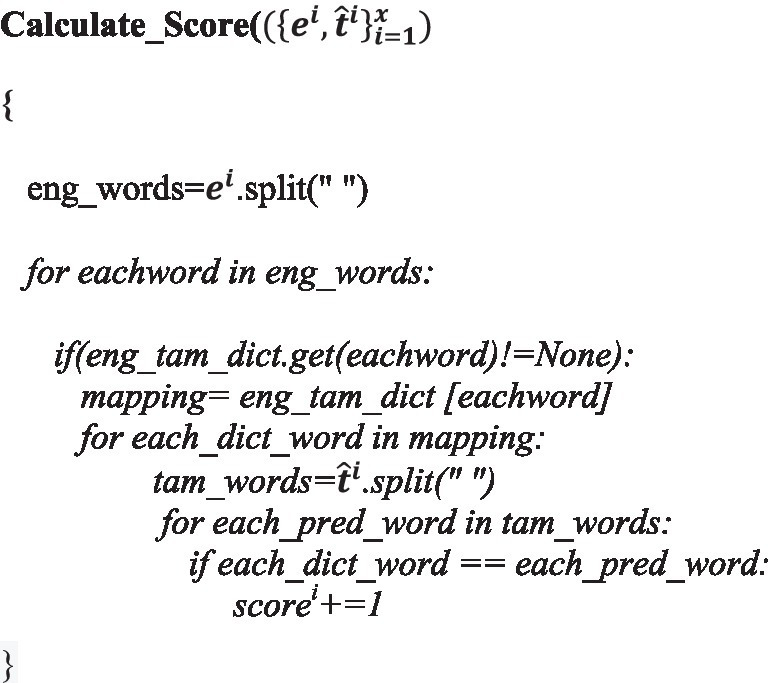


where

- ${\left\{e^{i},{\hat{t}}^{i}\right\}}_{i=1}^{x}$ is the list of *x* English monolingual sentences and their outputs obtained using the baseline NMT
- *eng_tam_dict* is the dictionary that maps each English word to all possible Tamil words of the training data with the same meaning
- *scores* is a list of *x* scores for the *x* sentences, where each score represents the count of the number of words in the dictionary that match with the words of the translated Tamil sentence.

#### ALGORITHM 3 Incremental training with SMT integration


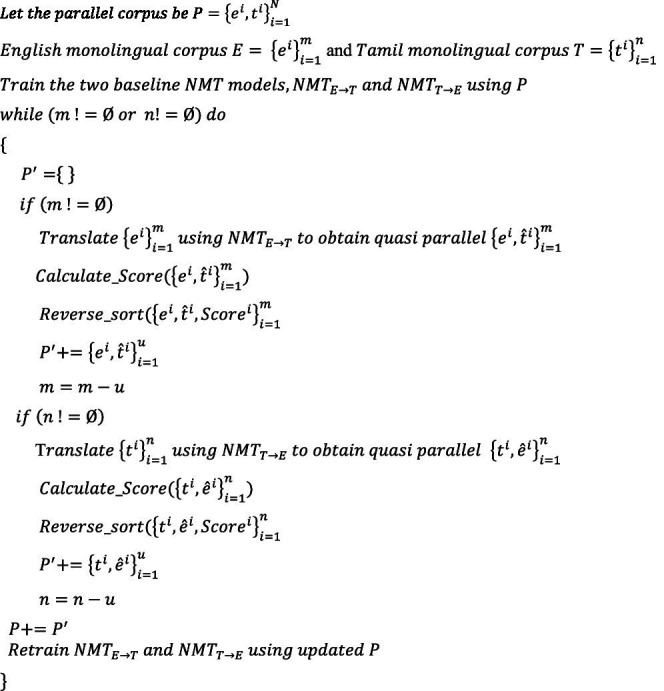


A score of 2/3 for the first English input sentence indicates that two words from the dictionary match with the model output. This infers that the model variant III selects sentences 1 and 2 as the two best translations out of the three samples, as the scores for these two sentences are the highest. Toward the end of the incremental training phase, the suboptimal NMT models of the previous phase are transformed into well-trained models and contribute significantly to performance improvement.

### 3.4 Model variant IV—baseline NMT + SMT-integrated incremental training + beam search

The output of the Softmax layer in the NMT model is a list of probabilities for each time step in the target sentence. These probabilities should be decoded into words from the vocabulary of the target language. Greedy search decoding chooses the word with the highest probability at any time step and outputs that single best word. Although a greedy method has the advantage of being incredibly rapid, it considers each position in isolation. After identifying the best word for a particular position, the words before or after that are not examined. Consequently, quality of the final output sequences may be far from ideal. Due to the downside of the greedy search algorithm, a beam search decoding technique for sequence-to-sequence models has been introduced for neural machine translation. Beam search decoding is a popular algorithm for decoding sequences of words in many natural language processing tasks, such as machine translation and text summarization. Instead of picking a single best word, it picks the *k* best words from the previous position and finds the probabilities of the combination of each of the *k* words with the word in the current position. This yields better results than the Greedy search. The hyperparameter *k* is referred to as the Beam width. The beamwidth *k* is used to control the trade-off between accuracy and speed. A higher value of *k* leads to more accurate results but slows down the decoding process; a lower value of *k* leads to faster but less accurate results.

**Figure 2:**
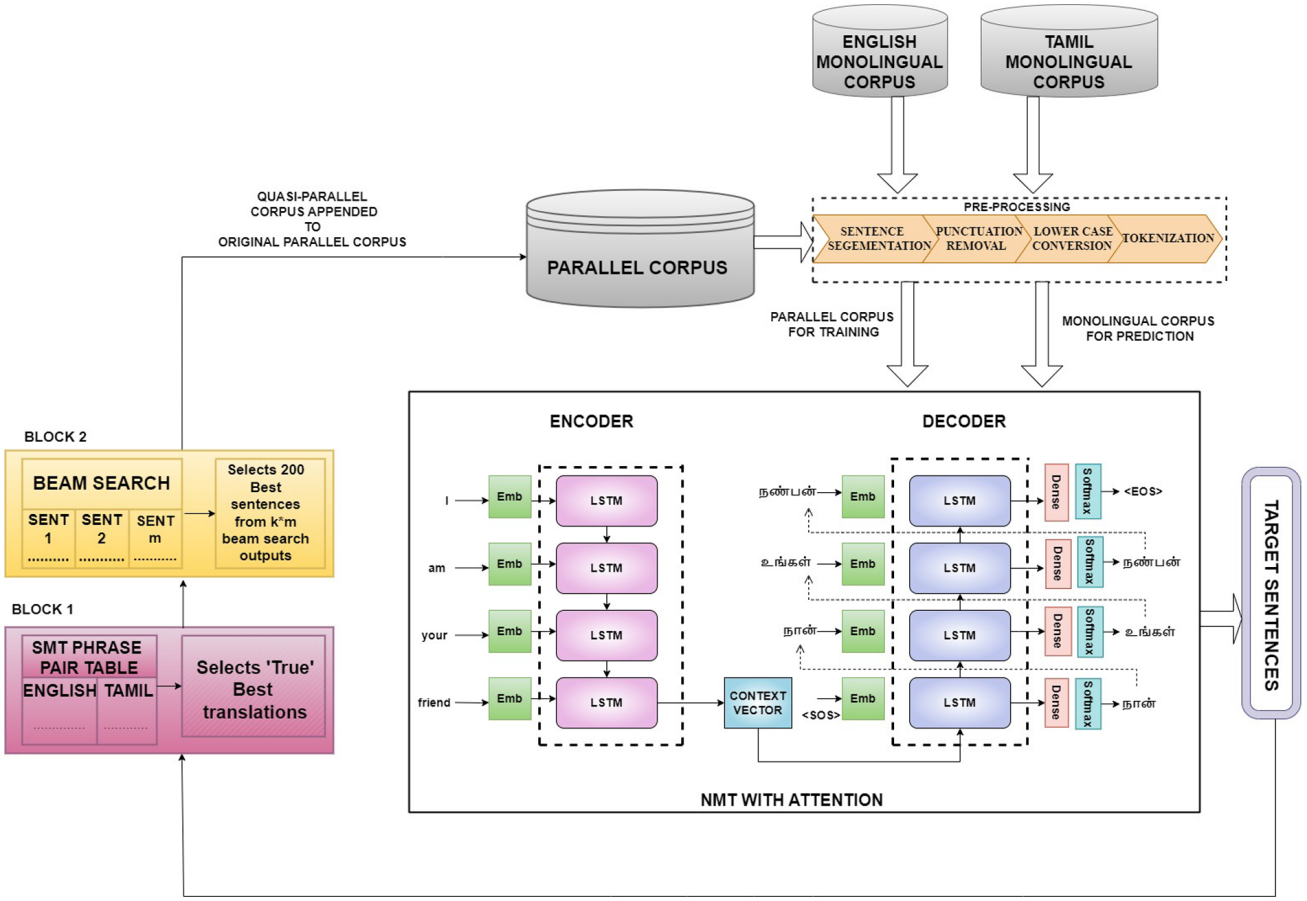
Comprehensive Representation of the Four Model Variants—Model Variant IV comprises all the components in the framework, including Block 1 and Block 2. Model Variant III comprises all the components in the framework except Block 2. Model Variant II is obtained by excluding both Block 1 and Block 2 that simply trains the NMT incrementally with random sampling. Model Variant I is the baseline NMT that includes only the encoder–decoder block.

For a single sentence of the source language, beam search decoding predicts *k* best sentences of the target language. Table 5 elucidates the score evaluation for the *k* beam search outputs of the three sample input sentences. Scores for the first English input sentence [1/3,1,1] show that only a single word from the dictionary matched with the first beam search output, whereas three words from the dictionary match with the other two outputs. There is a high probability that model variant IV reports only error-free translations such as sentences (1b) or (1c) and (2c) as the two best translations, unlike the model variant III that reports a few partially correct translations in the list of best translations. Integrating SMT with beam search outputs helps to identify more accurate translations. Figure 2 portrays the holistic view of all four model variants with exclusive blocks to represent the salient features of each variant.

**Table 5:** Score calculation for *k* beam search outputs of three sample sentences to select the two best translations.

English sentence	Tamil translated ‘k’ sentences (*k* = 3)	Score	Best two translations
1. I went to school	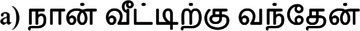	1/3 = 0.33	Sentences 1b or 1c and 2c
	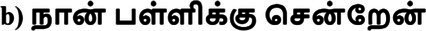	**3/3 = 1**	
	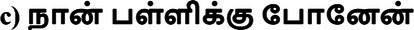	**3/3 = 1**	
2. He told a story	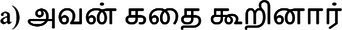	3/4 = 0.75	
	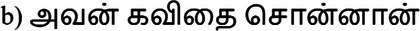	2/4 = 0.5	
	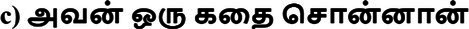	**4/4 = 1**	
3. Teacher came to the classroom	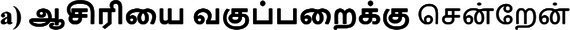	**2/3 = 0.66**	
	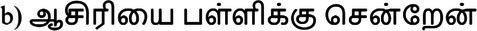	1/3 = 0.33	
	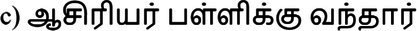	2/3 = 0.66	

Bold values (as in 1b or 1c) in the denominator represent the total number of words in the translated output. Numerator represents the number of words in the translated output that matched with the SMT dictionary.

## 4 Experimental setup and corpora

The proposed hybrid attention-based NMT-SMT model is implemented using ColabPro+ with an NVIDIA A100 Tensor Core GPU (Ampere architecture) and NVIDIA K80 GPU card

Number of CUDA cores: 6912

Memory: 40/80 GB HBM2e (high-bandwith Memory)

Memory bandwidth: 1.6/2 TB/s

FP32 performance: 19.5 TFLOPS

NMT implementation: encoder–decoder model consisting of an embedded layer, bidirectional LSTM layers, and a Softmax layer.

SMT implementation: GIZA++ tool.

Model evaluation metrics: BLEU (Papineni et al., 2002) and METEOR (Lavie and Denkowski, 2009). Higher scores indicate better translations.

Training period: 4 weeks

**Figure 3:**
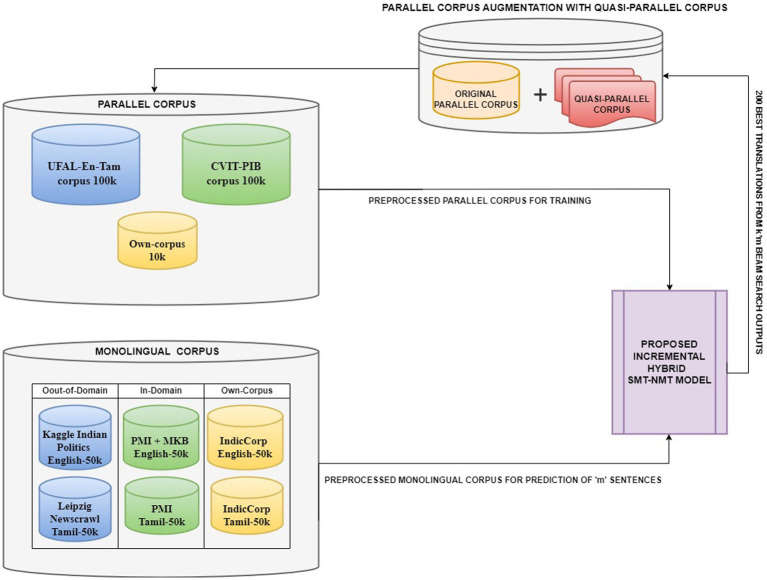
Data generation for the proposed hybrid model (The original and small, parallel corpus is augmented incrementally with the quasi-parallel corpus produced as output by the hybrid model in each iteration. This repeating cycle of data augmentation and then prediction after retraining the model with the augmented data generates a large-quasi-parallel corpus, adequate to improve the translation performance of the model for low-resource languages).

For the language pair English–Tamil, there are not many open-source bilingual corpora. CVIT-PIB (Siripragada et al., 2020) of 115 k parallel sentences obtained from online publicly available sources, UFAL EnTam-v2.0 (Ramasamy et al., 2012) with a focus on the movie and news industries and bible data amounting to 169.8 k parallel sentences are used for the empirical study. In addition, our own corpus of approximately 9.9 k parallel sentences is created from educational websites. For our experiments, we select a subset of 100 k parallel sentences from these bilingual resources. This curated dataset ensures a balanced representation across different domains while maintaining a manageable size for our empirical study. For the English monolingual dataset, IndicCorp (Kakwani et al., 2020), Kaggle Indian Politics News^1^ (online sources), and Mann-Ki-Baat^2^ in English (available online) are used. For the Tamil monolingual dataset, PMI dataset (Haddow and Kirefu, 2020) with 91 k sentences, Leipzig Newscrawl^3^ with 300 k sentences (online sources), and IndicCorp with 31.5 M and Mann-Ki-Baat in Tamil with 5.7 k sentences (available online) are used. The PM India corpus and the MKB (Mann-Ki-Baat) corpus contain the Indian Prime Minister’s address to Indian citizens, which is manually translated into many Indian languages. Despite the vastness of these monolingual resources, we limited our trials to a subset of approximately 50 k sentences from each English and Tamil monolingual corpora. Figure 3 picturizes the data generation process for the proposed hybrid model. This approach allows us to maintain the computational cost and still leverage the diversity of the available data. Table 6 lists the various corpora and their sizes.

**Table 6:** Parallel and monolingual corpora size.

PARALLEL CORPUS	
CVIT-PIB	119 K
UFAL EnTam	169 K
Own corpus	10 K
Tamil—MONOLINGUAL CORPUS	
PMI	91 K
Leipzig Newscrawl	300 K
IndicCorp	31.5 M
English—MONOLINGUAL CORPUS	
IndicCorp	54.3 M
PMI + MKB	90 k
Kaggle Indian Politics News	300 K

The four different model variants considered for the experimental study are baseline NMT-ATTN (attention mechanism), baseline NMT-ATTN + random sampling-based incremental training with monolingual corpus, baseline NMT-ATTN+ SMT-integrated incremental training, and baseline NMT-ATTN + SMT-integrated incremental training + beam search. These four approaches are implemented on three different categories of corpora.

- **Out-of-domain corpus**: UFAL EnTam is used as parallel corpus; Kaggle Indian Politics News and Leipzig Newscrawl are used as monolingual corpora for English and Tamil, respectively.
- **In-domain corpus**: CVIT-PIB is used as parallel corpus; MKB + PMI and PMI are used as monolingual corpora for English and Tamil, respectively.
- **In-domain own corpus**: Own corpus crawled from several websites (300-English-Sentences-With-Tamil-Meaning, 2023), (Tamil-Sentences-and-Phrases, 2023), and (Interrogative-Sentence, 2023) is used as parallel corpus; IndicCorp is used as monolingual corpora for English and Tamil, respectively.

## 5 Results and discussion

**Figure 4:**
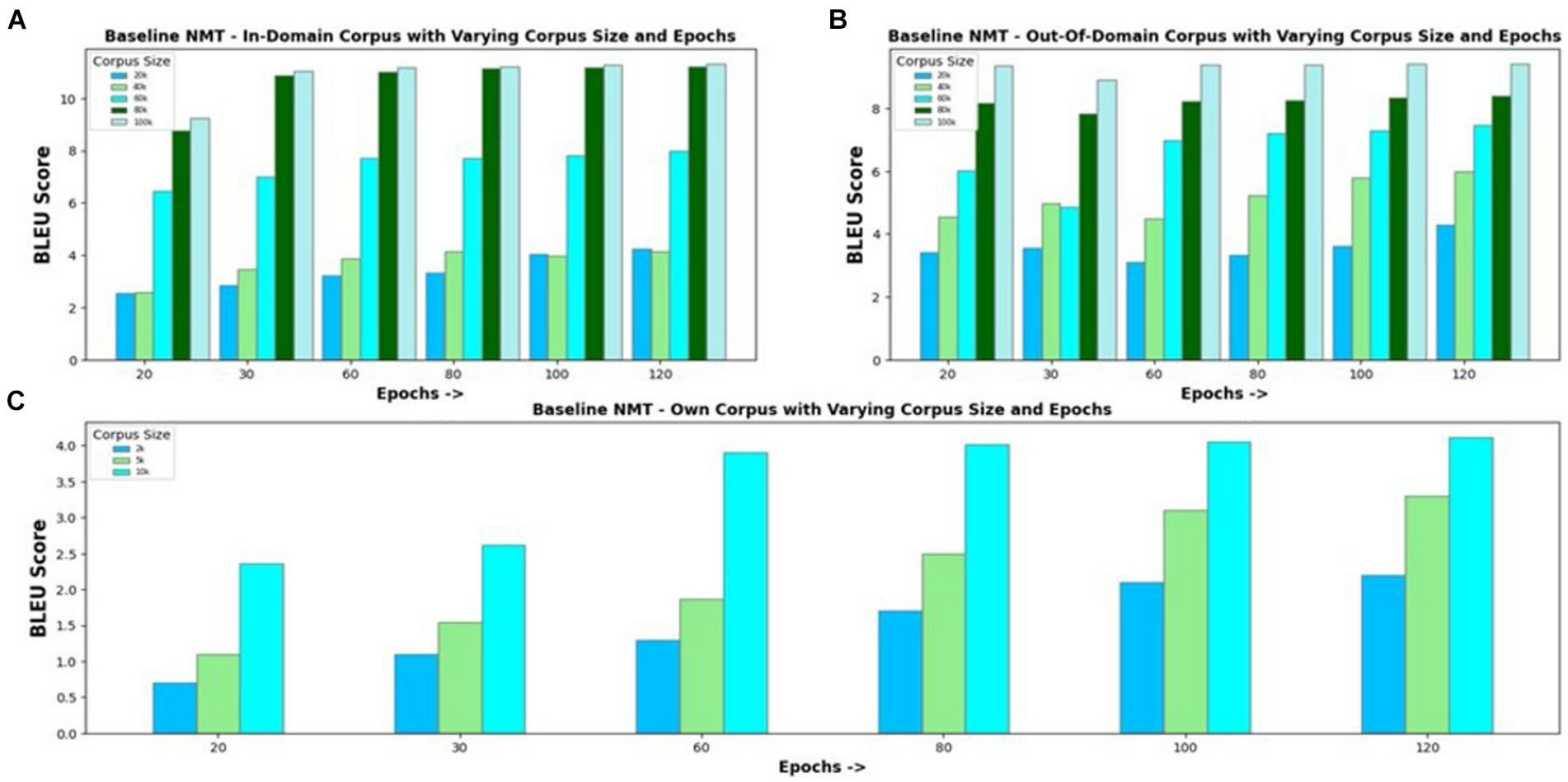
Baseline NMT with varying corpus size and epochs for **(A)** In-Domain Parallel Corpus; **(B)** Out-of-Domain Parallel Corpus; **(C)** Own Corpus.

NMT model constructed with an encoder–decoder and attention mechanism is used as the baseline to evaluate the proposed model’s performance. The baseline model is experimented with in-domain and out-of-domain corpora and with own corpus. Figure 4A visualizes the BLEU scores obtained for the Eng-to-Tam model when trained with an in-domain parallel corpus of varying sizes ranging from 10 k to 100 k and for different epochs. It can be seen that the translation performance increases with an increase in the training data size. Increasing the number of epochs provides comparatively better results for small corpus sizes. However, for corpus sizes greater than 30 k, increasing the number of epochs beyond 30 simply increases the training time without a proportionate improvement in accuracy. For this reason, the number of epochs is set to 30 in further investigations. Experimental results of the model trained with out-of-domain corpus and own corpus are shown in Figures 4B,C, respectively. The maximum BLEU score values obtained for out-of-domain corpus of size 100 k and own corpus of size 10 k are 9.42 and 4.12, respectively, which are comparatively less than that of in-domain corpus. Except for the difference in BLEU score values, other results are analogous to in-domain corpus, showing no remarkable improvement with increase in the number of epochs.

**Figure 5:**
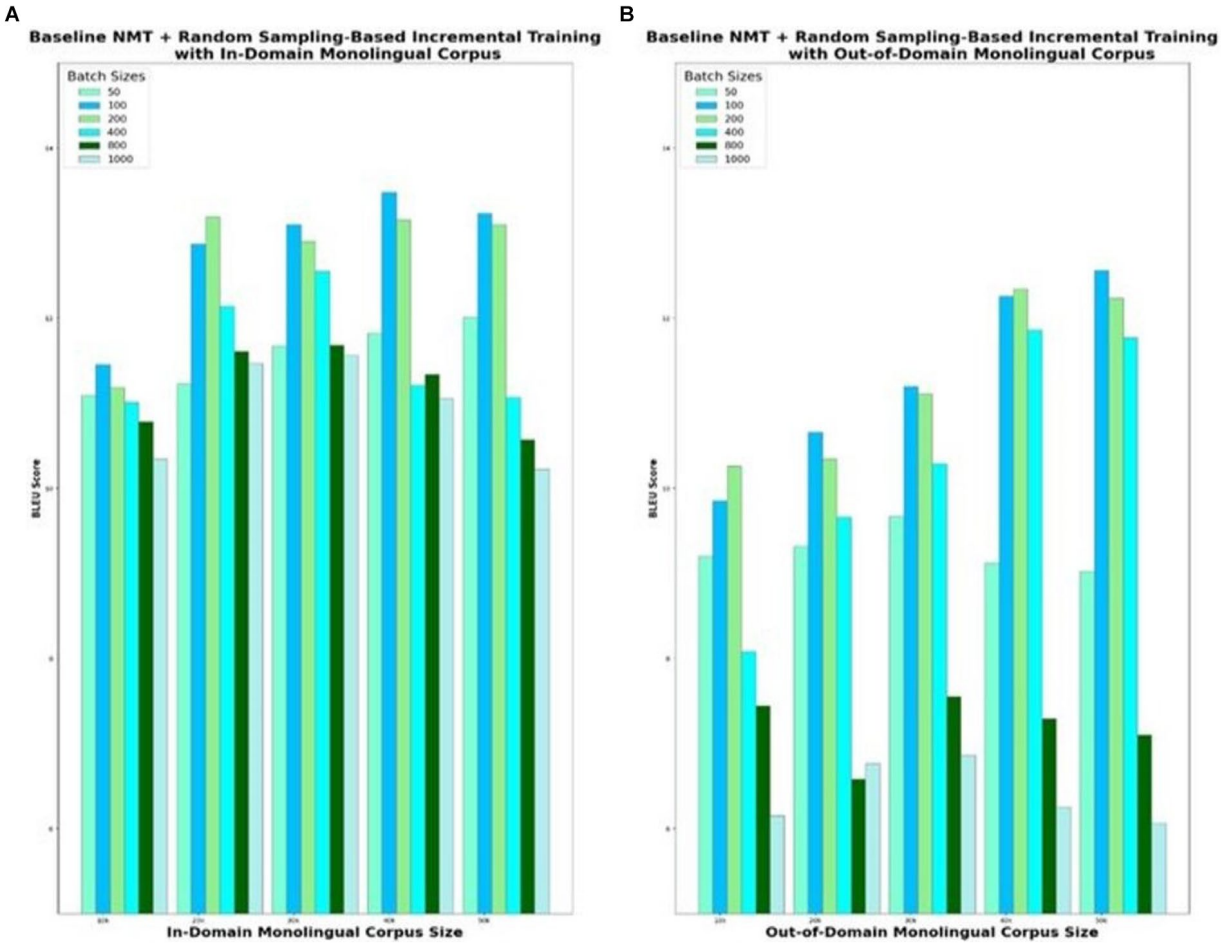
NMT with random sampling-based incremental training of monolingual corpus with varying set sizes. **(A)** In-Domain Monolingual Corpus; **(B)** Out-of-Domain Monolingual Corpus.

The baseline NMT augmented with random sampling-based incremental training is the second model variant that is evaluated. For experimental purposes, the baseline NMT is trained with the in-domain parallel corpus of 100 k sentences and integrated with incremental training. Incremental training is carried out with different sizes of monolingual corpus, ranging from 10 k to 50 k and different sizes of batches that are predicted in each iteration, ranging from 50 to 1,000. To begin with, the 10 k English monolingual corpus is split into multiple batches with each batch consisting of 50 randomly sampled sentences and fed as input to the Eng-to-Tam model. Similarly, 50 sentences from the Tamil monolingual corpus are provided as input to the Tam-to-Eng model. The input and output sentences of these two models taken together contribute to a total of 100 parallel sentences. Training repeats with this additional quasi-parallel data, and the improved models predict for the next batch of 50 sentences. BLEU score is recorded for the model, which is incrementally trained with the entire 10 k corpus in batches of size 50. In a similar way, the model is empirically tested for varying batch sizes ranging from 50 to 1,000 and the BLEU scores are plotted in Figure 5. The smaller the batch size, the better the accuracy, but at the cost of more training time. Analyzing the optimal trade-off between accuracy and time is vital to the success of incremental training. Based on the evaluation done for various monolingual corpus sizes from 10 to 50 k, increasing the batch size beyond 100 degrades the translation quality significantly. Batch sizes lesser than 100 offer trivial benefits in terms of translation performance but with adverse effects on the training time. Similar observations are made for out-of-domain corpus as well, with the best possible BLEU score of 12.56 for batch size 100, when the models are incrementally trained with 50 k monolingual corpus.

**Figure 6:**
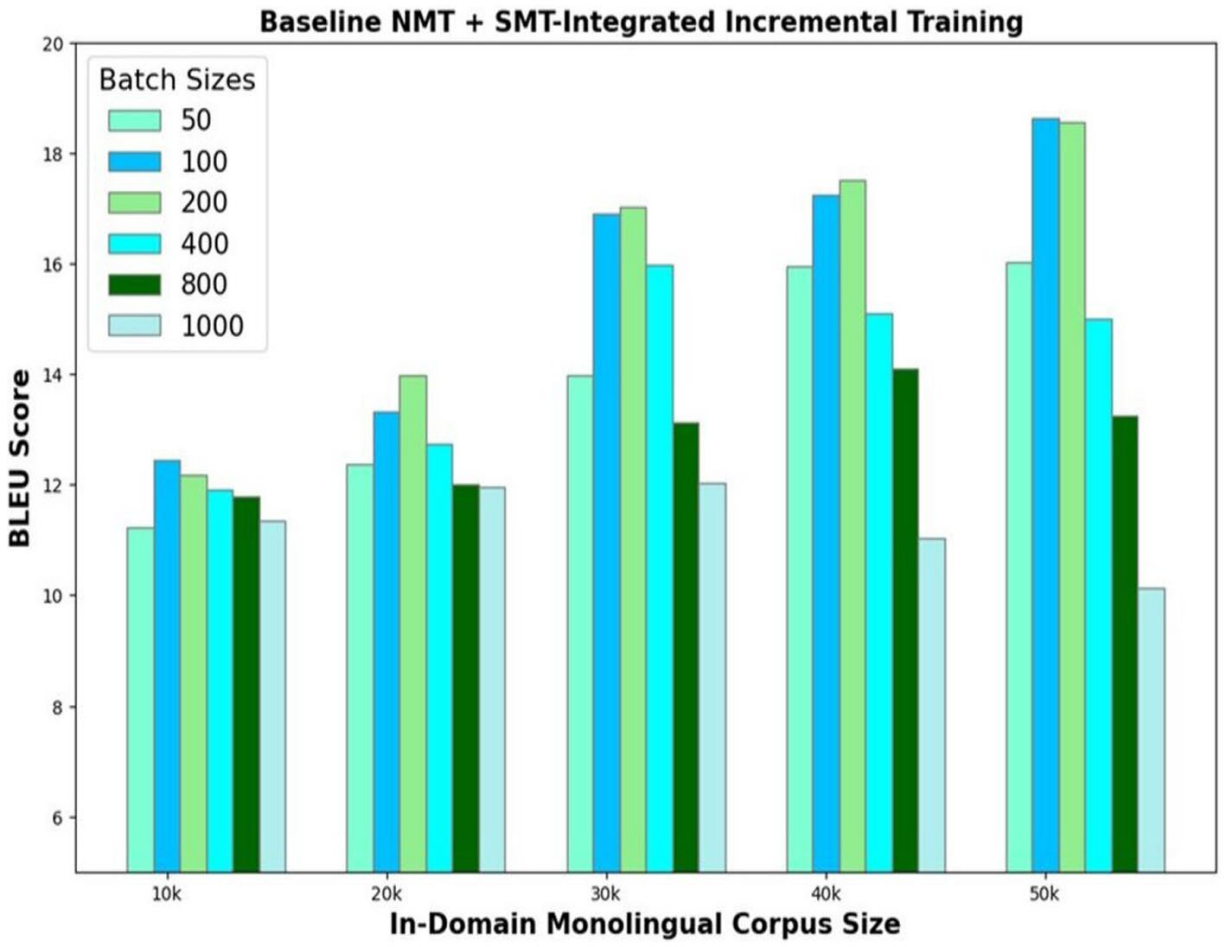
NMT with SMT-integrated incremental training of In-Domain monolingual corpus.

The third model variant exploits the advantages of both NMT and SMT. The basic encoder–decoder framework that is initially trained with a 100 k parallel corpus translates the 50 k In-Domain Monolingual corpus. The translations are evaluated using the SMT phrase-pair table generated using GIZA++. The count of the number of words in the SMT phrase-pair dictionary, which match with words of the corresponding model outputs, is found. Based on the count of the matching words, the best translations are identified and added to the parallel corpus. Experiments are conducted to determine the optimum number of best translations *u* to be selected in each iteration. The model’s performance is evaluated by choosing varying number of best translations ranging from 50 to 1,000. The BLEU scores of the NMT with SMT-integrated incremental training of the In-Domain Monolingual corpus size of 50 k are illustrated in Figure 6. Results show that the optimal value for *u* is 200 as the model yields the best translation accuracy without compromising the training speed if 200 best translations are selected and added to the parallel corpus, in each iteration. Model accuracy dwindles for *u* values greater than 200, and its learning speed reduces for *u* values less than 200.

The proposed model variant IV incorporates a beam search decoding strategy to reduce the errors further. The model trained with a parallel corpus of various sizes and a 50 k monolingual corpus is considered for benchmark evaluation. Beam search selects the *k* best possible translations for each sentence. To determine the optimum beam width, different values of *k* spanning from 2 to 20 are explored.

**Figure 7:**
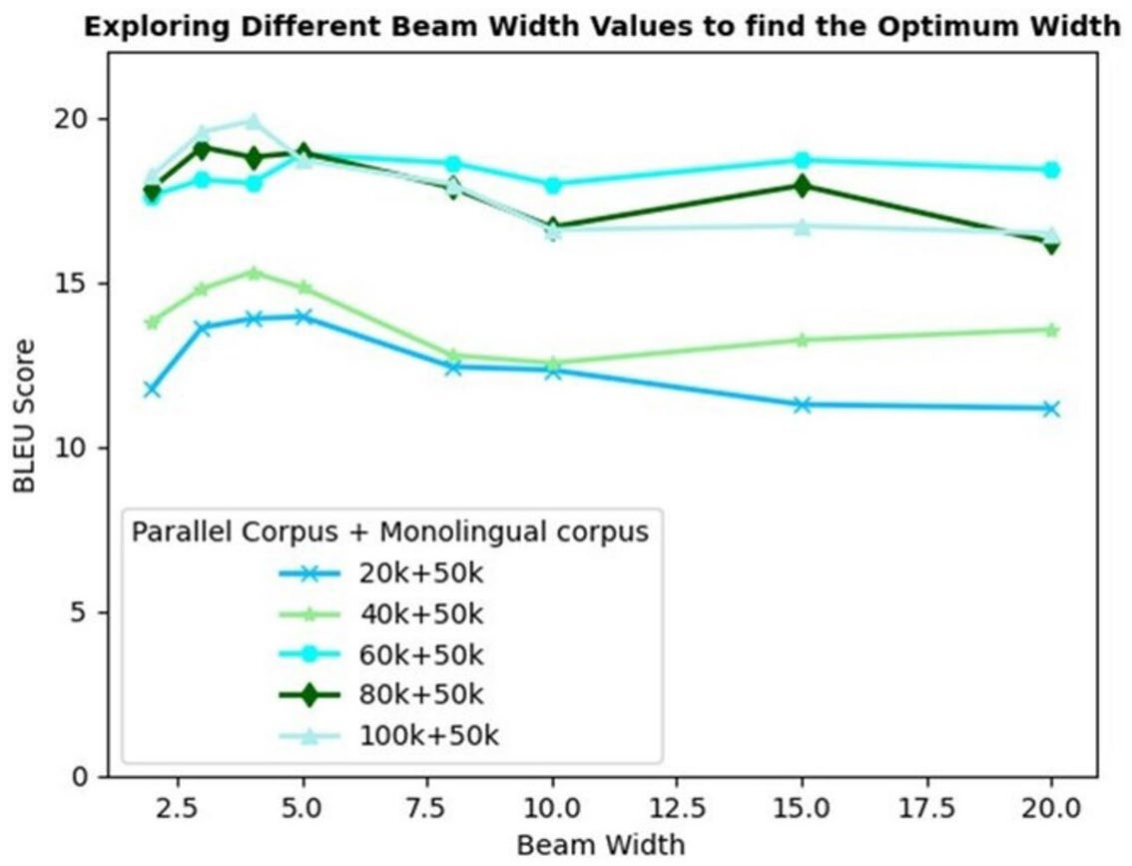
BLEU scores of varying corpus sizes and beam width.

Figure 7 depicts the trend of the BLEU scores recorded for varying corpus sizes and beam width values. The trendline demonstrates a steady rise in the BLEU score for up to a beam width of 5, on average. Increasing the beam width beyond 5 results in poor translation quality for most of the corpus sizes experimented. This is owing to the low brevity penalty for larger beam widths. Equation 6 implies that Brevity Penalty (BP) is applied to those machine translations that are shorter than the reference translations which is given by


(6) $$BP=\min\left\{e^{\left(1-1/lr\right)},1\right\},\text{with length ratio}\;\left(lr\right)=\;|y|/|y*|$$

Here, |y|and|y * | represent the generated translation length and reference length, respectively.

Increased beam width provides an option to explore more candidates with more flexibility in choosing multi-word phrases, thus resulting in shorter translations. This decreases|y|, causing ripple effects on the length ratio and brevity penalty (Spero, 2019; Yang Y. et al., 2018).

A beam width of 4 yields the highest BLEU score of 19.90 for 100 k parallel +50 k monolingual corpus. However, a beam width of 3 is found to be optimal with equivalently good results but without compromising the decoding speed. Incremental training combined with SMT phrase pairs table and beam search decoding evolves as the best model, recording the highest BLEU scores of 19.56 and 23.49 in Eng-to-Tam and Tam-to-Eng directions, respectively.

**Figure 8:**
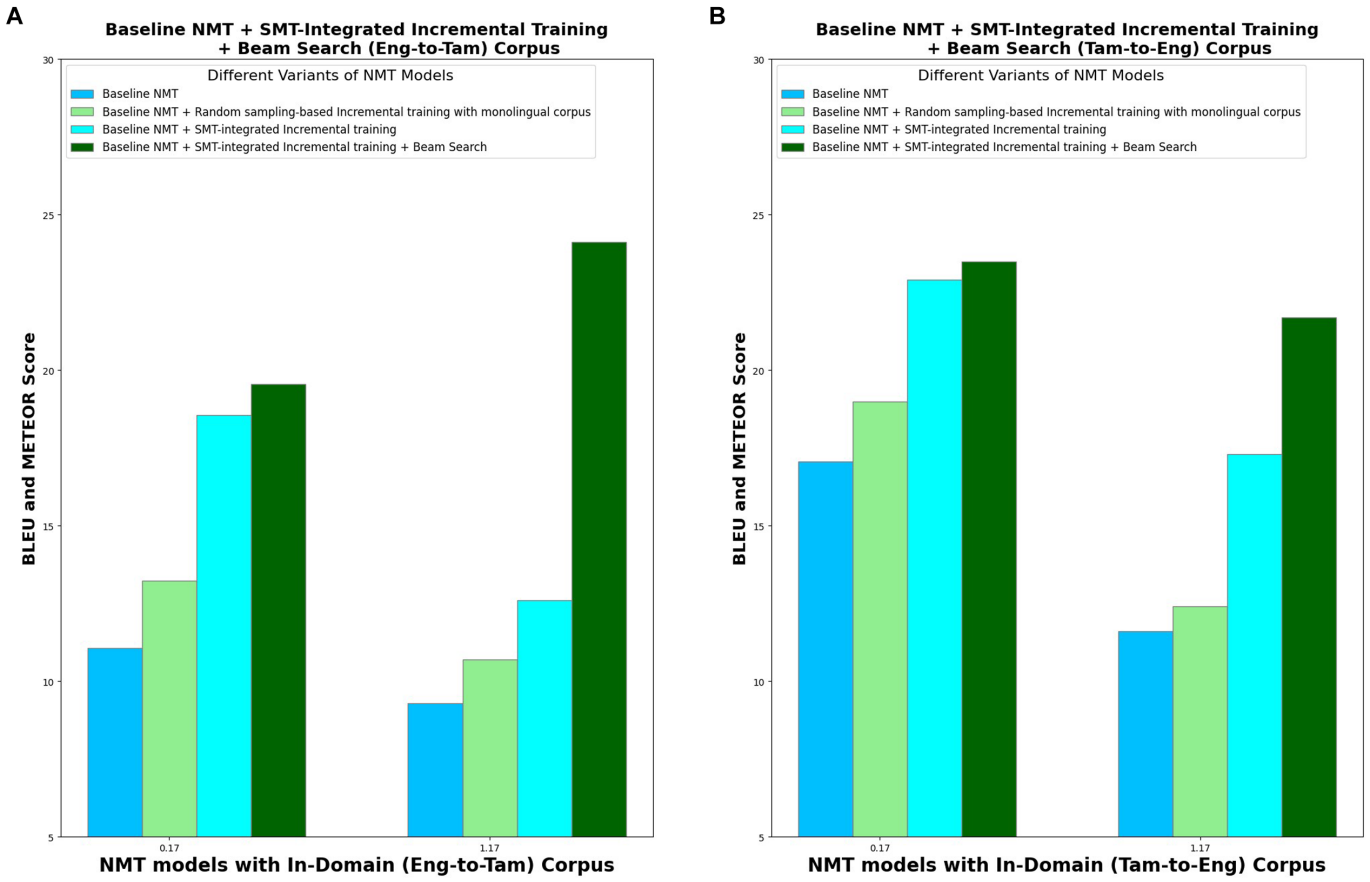
Comparative analysis of four NMT model variants in terms of BLEU and METEOR scores for in-domain. **(A)** Eng-to-Tam corpus and **(B)** Tam-to-Eng corpus.

In order to analyze the performance of the four model variants, METEOR score is also computed, in addition to the BLEU score. This is because BLEU considers only the precision for evaluation whereas METEOR considers both precision and recall, with recall weighted 9 times more than precision. This better correlates with human evaluation. Table 7 lists the BLEU and METEOR scores of the various model variants in both the directions (Eng-to-Tam and Tam-to-Eng), and Figure 8 picturizes the comparative analysis of the four model variants. With random sampling-based incremental training, BLEU scores increase by 19% in Eng-to-Tam translation and 11% in Tam-to-Eng translation which is significantly lesser than the 67 and 34% increase obtained with SMT-integrated incremental training in the two directions. From Figure 7, it is apparent that implementing the beam search encoding technique in addition to SMT integration contributes for a further increase in the translation performance, albeit marginal. This is for the reason that beam search helps to select the most appropriate translation, thus resulting in the BLEU scores of 19.56 and 23.49 in Eng-to-Tam and Tam-to-Eng translations, respectively.

**Table 7:** BLEU and METEOR scores of the various NMT model variants.

Different variants of the NMT model	Eng-to-Tam		Tam-to-Eng	
	BLEU	METEOR	BLEU	METEOR
Baseline NMT	11.06	9.3	17.06	11.6
NMT + incremental training (random sampling without replacement)	13.23	10.7	18.98	12.4
NMT + incremental training + SMT phrase pair	18.56	12.6	22.91	17.3
NMT + incremental training + SMT phrase pair + beam search (*k* = 3)	19.56	24.12	23.49	21.7

A steady increase is seen in the METEOR score values, from baseline NMT to model variant IV. METEOR results attest to the best performance of the model variant IV, and a higher value of METEOR than BLEU for Eng-to-Tam translation shows that the potential of the model to generate more human-like translations.

In order to prove the supremacy of the proposed model over the methods suggested in the literature, results of model variant IV are compared with the BLEU scores of two very similar methods—one implemented by Zhang et al. (2017) for English-German translation (en-de) and the other implemented by Lample et al. (2018) for forward–backward translation of English, Roman, and Russian languages (en-ro & ro-en and en-ru & ru-en). From Table 8, it is seen that the BLEU scores of the proposed model for en-ta translation is better than those of en-de and en-ru translation by the existing methods. However, the backward translation performance of the proposed model (ta-en) is less than (ro-en) translation performance of the existing method, it is still better than (ru-en) translation. This proves the suitability of the proposed model variant IV for low-resource machine translation tasks.

**Table 8:** BLEU score comparison of the proposed model with existing approaches.

Models		source→target	BLEU score
Zhang et al. (2017)	PBMT + rerank (development sets)	en→de	14.23
		en→fr	28.86
	PBMT + rerank (test sets)	en→de	15.89
		en→fr	29.77
Lample et al. (2018)	PB-SMT + NMT	en→ro	25.13
		en→ru	13.76
		ro→en	23.90
		ru→en	16.62
	NMT + PB-SMT	en→ro	21.95
		en→ru	10.14
		ro→en	23.73
		ru→en	12.62
Proposed model [Variant IV—NMT+ incremental training + SMT phrase pair + beam search (*k* = 3)]		**en**→**ta**	**19.56**
		**ta**→**en**	**23.49**

The highlighted numbers indicate the BLEU scores of the proposed model for Eng-to-Tam and Tam-to-Eng translations respectively.

### 5.1 Practical implications

In the realm of academic linguistics, machine translation serves as a vital tool for both language learners and instructors. It is crucial for the exchange of knowledge, ideas, and information, facilitating effective communication and transcending international barriers through natural language processing. In this direction, the proposed model can be used for the translation of other low-resource languages, in a similar way. This is significant because better translations for low-resource languages will help to go a long way in achieving the goal of conversation using natural language as the next-generation human–computer interface. In addition, it will enable the existing data-driven models to extend into many new applications that require natural language processing. Owing to the incremental training of the model, the model can be deployed and explored in resource-limited environments.

## 6 Conclusion

A novel NMT-SMT hybrid framework is proposed which attempts to leverage the benefits of incremental training and beam search decoding, to provide more natural translations for low-resource languages. Experiments are conducted with varying sizes of parallel in-domain, out-of-domain, and own corpora to study the applicability of the model in a wider perspective. Randomly translating the monolingual sentences in batches, with optimal batch size, and using them to incrementally train the baseline NMT contributes moderately toward the elevated performance of the model. Intuitively, using only the best translations to retrain the model in the subsequent iterations will amplify the power of incremental training manifold. In an effort to select the “true” best translations, the phrase table generated using GIZA++ as a part of SMT is reliably integrated with incremental training. Using the SMT phrase pair tables to select the best translations, results in a phenomenal increase of approximately 67 and 34% in the BLEU scores for Eng-to-Tam and Tam-to-Eng translations. This is owing to the fact that the initial small parallel corpus is gradually transformed into a large quasi-parallel corpus. In addition, the proposed model implements beam search decoding that improves the results further. The customized model (NMT+ SMT-integrated incremental training + Beam search) produces contextually appropriate translations, recording the highest BLEU scores of 19.56 and 23.49 in Eng-to-Tam and Tam-to-Eng translations, respectively, surpassing the results of the other model variants. The obtained results are equivalent to that of high-resource languages, especially for in-domain data.

### 6.1 Academic contributions

The findings of this research study add to the literature on neural machine translation for low-resource languages. Our idea of NMT-SMT hybrid model opens up new avenues for the state-of-the-art research on multimodal translation. The model is capable of providing high-quality text translations that can be improved further by using visual cues and audio modality (Futeral et al., 2024; Sulubacak et al., 2020).

Conversational AI market encourages low-resource language researchers and content writers to extend their high-quality translations to more languages, develop more inclusive technologies and share localized knowledge and their content to generate large datasets (Costa-jussà et al., 2022). To this end, our research outcomes in terms of effective use of monolingual data to build high-quality parallel corpus and an amalgamation of SMT and NMT to build a better model contributes to the exploration of more under-resourced languages. It can also allow for easy translation of literary text of low-resource languages.

Existing models for the translation of low-resource languages employ inappropriate methods to evaluate the NMT outputs (Zhang et al., 2018) and back-translation (Vyawahare et al., 2022) which does not generate high-quality, synthetic parallel corpus. The proposed model addresses these issues and improves the machine translation quality for low-resource languages, even with scarce parallel corpus.

- **Enhanced Translation Quality for Low-Resource Languages**: First, a more accurate and reliable machine translation model is built by integrating the best features of NMT and SMT. NMT works better for languages with significantly different word order and rich morphology and produces fast and more fluent translations; however, during the initial stage, when the parallel corpus volume is low or the corpus quality is low, outputs of SMT are more reliable.
- **Efficient Utilization of Monolingual Corpus**: Second, while attempting to use the monolingual corpus to augment the scarce parallel corpus, SMT is used to evaluate the translations of NMT and select the best translations with which the parallel corpus is augmented. Incremental training of the NMT with these best translations improves the translation performance of the model in successive iterations. SMT and incremental training have been proven to produce high-quality quasi-parallel corpus, as compared to back-translation and other techniques reported in the literature.
- **Reduced Training Time and Computational Complexity**: Incremental training avoids training the model from scratch but instead fine-tunes the model with quasi-parallel data. This significantly reduces the training time and computational complexity.
- **Efficient Navigation of Large Search Spaces**: With efficient values for beam width, beam search decoding empowers the model by selecting the best output sequence with a limited number of possible sequences to be explored.

### 6.2 Limitations

The model lags a little in performance for out-of-domain data and requires enhancements for the efficient handling of idioms, rare or OOV (Out-of-Vocabulary) words, and literary texts.

### 6.3 Future studies

As a future study, the performance of the proposed model in other low-resource languages can be investigated. New approaches can be researched to make the model efficiently handle idioms, rare or OOV (Out-of-Vocabulary) words, long sentences, and out-of-domain data. Cross-lingual transfer learning can be attempted to build translation models for low-resource languages from high-resource settings, by leveraging the fundamental similarities in the language structures. Exploring multilingual models and methods that require minimal training such as zero-shot translation will also prove to be useful in low-resource settings. In addition, crowdsourcing can be considered for augmenting the parallel corpus, as a step toward addressing the challenges of low-resource languages.

## Data Availability

The original contributions presented in the study are included in the article/supplementary material, further inquiries can be directed to the corresponding author.

## Footnotes

^1^ https://www.kaggle.com/xenomorph/indian-politics-news-2018

^2^ https://www.narendramodi.in/maan-ki-baat

^3^ http://cls.corpora.uni-leipzig.de/en/tam_newscrawl_2011

## Glossary

BLEU: Bilingual Evaluation Understudy
BP: Brevity Penalty
CVIT-PIB: Center for Visual Information Technology—Press Information Bureau
GAN: General Adversarial Networks
LSTM: Long Short-Term Memory
mBART: multilingual Bidirectional and Auto-Regressive Transformer
METEOR: Metric for Evaluation of Translation with Explicit ORdering
MKB: Mann Ki Baat corpus
NLP: Natural Language processing
NMT: Neural Machine Translation
NMT-ATTN: Neural machine translation—Attention mechanism
OOV: Out of vocabulary
PB-SMT: Phrase-Based Statistical Machine Translation
PMI: Prime Minister India corpus
SMT: Statistical Machine Translation
UFAL EnTam: University Institute of Formal and Applied Linguistics—English–Tamil corpus

## References

[ref1] 300-English-Sentences-With-Tamil-Meaning (2023). Available at: https://lifeneeye.com/spokeneng/basic-sentences/300-english-sentences-with-tamil-meaning/ (Accessed January 07, 2023).

[ref2] Almahairi A. Cho K. Habash N. Courville A., (2016). First result on Arabic neural machine translation. *arXiv* [Preprint] *arXiv:1606.02680*. (Accessed January 09, 2023).

[ref3] Artetxe M. Labaka G. Agirre E. Cho K., (2017). Unsupervised neural machine translation. *arXiv* [Preprint] *arXiv:1710.11041*. (Accessed February 03, 2023).

[ref4] Bahdanau D. Cho K. Bengio Y., (2014). Neural machine translation by jointly learning to align and translate. *arXiv* [Preprint] *arXiv:1409.0473*. (Accessed February 06, 2023).

[ref5] Banik D. Ekbal A. Bhattacharyya P. (2018). Machine learning based optimized pruning approach for decoding in statistical machine translation. IEEE Access. 7, 1736–1751. doi: 10.1109/access.2018.2883738

[ref6] Chen X. Li J. Wang H. (2019). Keyphrase enhanced diverse beam search: a content-introducing approach to neural text generation. IEEE Access 7, 72716–72725. doi: 10.1109/ACCESS.2019.2919974

[ref7] Cho K. Van Merriënboer B. Gulcehre C. Bahdanau D. Bougares F. Schwenk H. . (2014). Learning phrase representations using RNN encoder-decoder for statistical machine translation. *arXiv* [Preprint] *arXiv:1406.1078*. (Accessed January 03, 2023).

[ref8] Costa-jussà M. R. Cross J. Çelebi O. Elbayad M. Heafield K. Heffernan K. . (2022). No language left behind: scaling human-centered machine translation. *arXiv* [Preprint] *arXiv: 2207.04672*. (Accessed March 13, 2023).

[ref9] Edunov S. Ott M. Auli M. Grangier D., (2018). Understanding back-translation at scale. *arXiv* [Preprint] *arXiv:1808.09381*. (Accessed January 03, 2023).

[ref10] Fadaee M. Bisazza A. Monz C., (2017). Data augmentation for low-resource neural machine translation. *arXiv* [Preprint] *arXiv:1705.00440*. (Accessed January 13, 2023).

[ref11] Freitag M. Al-Onaizan Y., (2017). Beam Search strategies for neural machine translation. *arXiv* [Preprint] *arXiv:1702.01806*. (Accessed February 18, 2023).

[ref12] Futeral M. Schmid C. Sagot B. Bawden R., (2024). Towards zero-shot multimodal machine translation. *arXiv* [Preprint] *arXiv: 2407.13579*. (Accessed August 18, 2024).

[ref13] Gulcehre C. Firat O. Xu K. Cho K. Barrault L. Lin H. C. . (2015). On using monolingual corpora in neural machine translation. *arXiv* [Preprint] *arXiv:1503.03535*. (Accessed February 20, 2023).

[ref14] Haddow B. Kirefu F., (2020). PMIndia--A collection of parallel corpora of languages of India. *arXiv* [Preprint] *arXiv: 2001.09907*. (Accessed February 20, 2023).

[ref15] He J. Gu J. Shen J. Ranzato M. A., (2019). Revisiting self-training for neural sequence generation. *arXiv* [Preprint] *arXiv:1909.13788*. (Accessed March 01, 2023).

[ref16] He W. He Z. Wu H. Wang H. (2016). “Improved neural machine translation with SMT features” in Proceedings of the AAAI conference on artificial intelligence, vol. 30. doi: 10.1609/aaai.v30i1.9983

[ref17] Hong-Viet T. Van-Vinh N. Hoang-Quan N. (2021). Improving machine translation quality with denoising autoencoder and pre-ordering. J. Comput. Inf. Technol. 29, 39–56. doi: 10.20532/cit.2021.1005316

[ref18] Imamura K. Fujita A. Sumita E. (2018). “Enhancement of encoder and attention using target monolingual corpora in neural machine translation” In Proceedings of the 2nd workshop on neural machine translation and generation, ed. Birch A.. Melbourne, Australia: Association for Computational Linguistics, 55–63. doi: 10.18653/v1/W18-2707

[ref19] Interrogative-Sentence (2023). Available at: https://ilearntamil.com/interrogative-sentence/ (Accessed January 07, 2023).

[ref20] Jahier Pagliari D. Daghero F. Poncino M. (2020). Sequence-to-sequence neural networks inference on embedded processors using dynamic beam search. Electronics 9:337. doi: 10.3390/electronics9020337

[ref21] Jiao W. Wang X. Tu Z. Shi S. Lyu M. R. King I., (2021). Self-training sampling with monolingual data uncertainty for neural machine translation. *arXiv* [Preprint] *arXiv: 2106.00941* (Accessed February 22, 2023).

[ref22] Kakwani D. Kunchukuttan A. Golla S. Gokul N. C. Bhattacharyya A. Khapra M. M. . (2020). “IndicNLPSuite: monolingual corpora, evaluation benchmarks and pre-trained multilingual language models for Indian languages” In Findings of the Association for Computational Linguistics: MNLP 2020, 4948–4961.doi: 10.18653/v1/2020.findings-emnlp.445

[ref23] Koehn P. Och F. J. Marcu D. (2003). “Statistical phrase-based translation” in In 2003 conference of the North American chapter of the Association for Computational Linguistics on human language technology (HLT-NAACL 2003) (Stroudsburg, PA, USA: Association for Computational Linguistics), 48–54. doi: 10.3115/1073445.1073462

[ref24] Lamb A. Xie M., (2016). Convolutional encoders for neural machine translation. WEB download. Availabe at: www-cs.stanford.edu.

[ref25] Lample G. Ott M. Conneau A. Denoyer L. Ranzato M. A., (2018). Phrase-based & neural unsupervised machine translation. *arXiv* [Preprint] *arXiv:1804.07755*. (Accessed February 06, 2023).

[ref26] Lavie A. Denkowski M. J. (2009). The METEOR metric for automatic evaluation of machine translation. Mach. Transl. 23, 105–115. doi: 10.1007/s10590-009-9059-4

[ref001] Madasamy A. K. Hegde A. Banerjee S. Chakravarthi B. R. Priyadharshini R. Shashirekha H. (2022). “Overview of the Shared Task on Machine Translation in Dravidian Languages”. In: Proceedings of the second workshop on speech and language technologies for dravidian languages.. ed. Chakravarthi B. R.. 271–278. doi: 10.18653/v1/2022.dravidianlangtech-1.41

[ref27] Och F. J. Ney H. (2000). “Improved statistical alignment models.” Hong Kong: Association for Computational Linguistics. 440–447. doi: 10.3115/1075218.1075274

[ref28] Papineni K. Roukos S. Ward T. Zhu W. J. (2002). “Bleu: a method for automatic evaluation of machine translation” In Proceedings of the 40th annual meeting of the association for computational linguistics. Philadelphia, eds. Isabelle P. Charniak E. Lin D. Pennsylvania, USA: Association for Computational Linguistics, 311–318. doi: 10.3115/1073083.1073135

[ref29] Park C. Yang Y. Park K. Lim H. (2020). Decoding strategies for improving low-resource machine translation. Electronics 9:1562. doi: 10.3390/electronics9101562

[ref30] Qi Y. Sachan D. S. Felix M. Padmanabhan S. J. Neubig G., (2018). When and why are pre-trained word embeddings useful for neural machine translation? *arXiv* [Preprint] *arXiv:1804.06323*. (Accessed January 10, 2023).

[ref31] Ramasamy L. Bojar O. Žabokrtský Z. (2012). “Morphological processing for English-Tamil statistical machine translation” in Proceedings of the workshop on machine translation and parsing in Indian languages, 113–122.

[ref32] Sennrich R. Haddow B. Birch A., (2015). Improving neural machine translation models with monolingual data. *arXiv* [Preprint] *arXiv:1511.06709*. (Accessed February 12, 2023).

[ref33] Singh U. Goyal V. Lehal G. S. (2016). Urdu to Punjabi machine translation: an incremental training approach. Int. J. Adv. Comput. Sci. Appl. 7, 227–238. doi: 10.14569/IJACSA.2016.070428

[ref34] Singh S. M. Singh T. D. (2022). Low resource machine translation of english–manipuri: a semi-supervised approach. Expert Syst. Appl. 209:118187. doi: 10.1016/j.eswa.2022.118187

[ref35] Siripragada S. Philip J. Namboodiri V. P. Jawahar C. V., (2020). A multilingual parallel corpora collection effort for Indian languages. *arXiv* [Preprint] *arXiv:2007.07691*. (Accessed January 18, 2023).

[ref36] Spero M. (2019). Improved beam search diversity for neural machine translation with K-DPP sampling. Available at: https://web.stanford.edu

[ref37] Sulubacak U. Caglayan O. Grönroos S. A. Rouhe A. Elliott D. Specia L. . (2020). Multimodal machine translation through visuals and speech. Mach. Transl. 34, 97–147. doi: 10.1007/s10590-020-09250-0

[ref38] Sutskever I. Vinyals O. Le Q. V. (2014). Sequence to sequence learning with neural networks. Adv. Neural Inf. Proces. Syst. 27, 3104–3112. doi: 10.48550/arXiv.1409.3215

[ref39] Tamil Language (2023). Wikipedia. Available at: https://en.wikipedia.org/wiki/Tamil_language (Accessed June 16, 2023).

[ref40] Tamil-Sentences-and-Phrases (2023). Available at: https://www.learnentry.com/english-to-tamil/tamil-sentences-and-phrases/ (Accessed January 07, 2023).

[ref41] Vyawahare A. Tangsali R. Mandke A. Litake O. Kadam D. (2022). PICT@ DravidianLangTech-ACL2022: neural machine translation on dravidian languages. *arXiv* [Preprint]. *arXiv:2204.09098*. (Accessed April 28, 2023).

[ref42] Wang X. Lu Z. Tu Z. Li H. Xiong D. Zhang M. (2017). “Neural machine translation advised by statistical machine translation” in Proceedings of the AAAI conference on artificial intelligence. San Francisco, California, USA: AAAI Press (AAAI’17), 31, 3330–3336.

[ref43] Wang X. Tu Z. Zhang M. (2018). Incorporating statistical machine translation word knowledge into neural machine translation. IEEE/ACM Trans. Audio Speech Lang Process. 26, 2255–2266. doi: 10.1109/TASLP.2018.2860287

[ref44] Yang Z. Chen W. Wang F. Xu B. (2018). Unsupervised neural machine translation with weight sharing. *arXiv* [Preprint] *arXiv:1804.09057*. (Accessed April 28, 2023).

[ref45] Yang Y. Huang L. Ma M. (2018). Breaking the beam search curse: a study of (re-) scoring methods and stopping criteria for neural machine translation. *arXiv* [Preprint] *arXiv:1808.09582*. (Accessed March 15, 2023).

[ref46] Zhang Z. Liu S. Li M. Zhou M. Chen E. (2018). “Joint training for neural machine translation models with monolingual data” in Proceedings of the AAAI conference on artificial intelligence, vol. 32. doi: 10.1609/aaai.v32i1.11248

[ref47] Zhang J. Luan H. Sun M. Zhai F. Xu J. Liu Y. (2021). Neural machine translation with explicit phrase alignment. IEEE/ACM Trans. Audio Speech Lang. Process. 29, 1001–1010. doi: 10.1109/TASLP.2021.3057831

[ref48] Zhang J. Utiyama M. Sumita E. Neubig G. Nakamura S., (2017). Improving neural machine translation through phrase-based forced decoding. *arXiv* [Preprint] *arXiv:1711.00309*. (Accessed March 09, 2023).

